# Non-invasive strategy: Developing a topical IL-4Rα-specific nanobody for the treatment of allergic airway diseases

**DOI:** 10.1016/j.mtbio.2024.101148

**Published:** 2024-07-08

**Authors:** Taeyoung Ahn, Dong Hyuk Lee, GeunAh Kim, JiHyun Kim, Joon-Sang Park, Hyung-Ju Cho, Joo Young Kim

**Affiliations:** a Department of Pharmacology and Brain Korea 21 Project for Medical Science, Yonsei University College of Medicine, Seoul, Republic of Korea; b Woo Choo Lee Institute for Precision Drug Development, Seoul, Republic of Korea; c Department of Computer Engineering, Hongik University, Seoul, 121-791, Republic of Korea; d Department of Otorhinolaryngology, Yonsei University College of Medicine, Seoul, Republic of Korea

**Keywords:** Allergic rhinitis, Interleukin-4 receptor α, Nanobody, *In-silico* affinity maturation, Non-invasive treatment

## Abstract

Inhibiting IL-4 and IL-13 are critical cytokines that induce the pathogenic responses of allergic airway diseases. Currently, monoclonal antibodies targeting IL-4Rα are administered subcutaneously to treat eosinophilic rhinosinusitis and allergic asthma. However, these treatments have several drawbacks. To address these issues, we have developed a novel IL-4Rα-targeting nanobody designed for non-invasive delivery to local inflammatory sites in allergic airway diseases. H5, selected via the ribosomal display applied screening from synthetic nanobody library, underwent dimerization and *in-silico* affinity maturation using AlphaFold2 and GROMACS resulting in a substantial/dramatic enhancement of its binding affinity. H5 effectively controlled inflammatory markers such as *MUC5AC*, *CCL26*, and *FOXJ1* in human nasal epithelial cells (HNECs) by inhibiting IL-4 and IL-13 signaling. The bivalent form of H5 showed efficacy in easily accessible cells, such as multi-ciliated cells, while the monovalent variant targeted hard-to-reach cells, such as basal cells of HNECs. In summary, we developed a nanobody that could effectively inhibit inflammatory signaling in HNECs via intranasal administration, showing promise as a non-invasive rhinitis treatment.

## Highlights

• H5, a nanobody for IL-4 receptor α was discovered using synthetic nanobody library screening.
• The IL-4 receptor α-binding affinity of H5 was improved by *in-silico* affinity maturation along with dimer formation.
• The high penetrability of monomeric H5 more affects basal cells located deep inside the cell layer.
• The high affinity of bivalent H5 greater affects ciliated cells nearby the apical side.

## Graphical abstract

**Figure:**
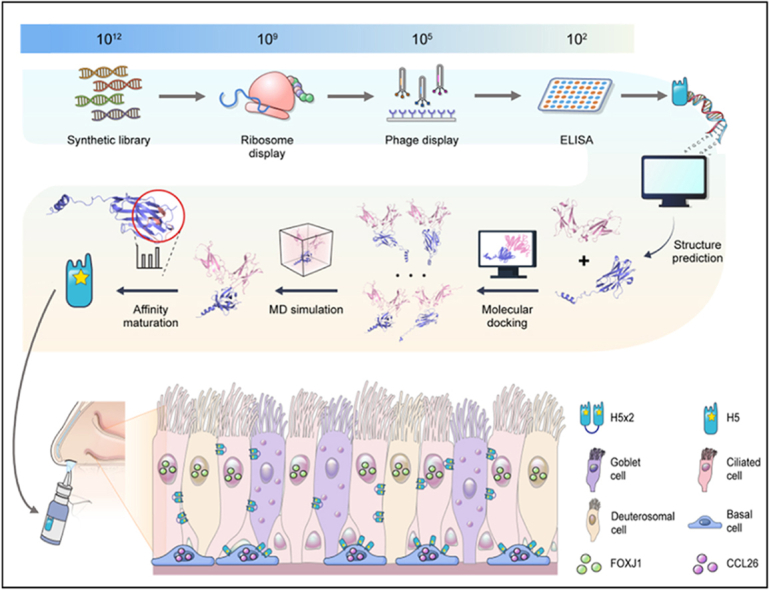

## 1 Introduction

Allergic rhinitis, eosinophilic rhinosinusitis, Rhinitis, asthma, and atopic dermatitis are allergic diseases controlled type 2 inflammatory cytokine such as IL-4 and IL-13 [1]. In type 2 inflammation, IL-4Rα (IL-4 Receptor alpha) associates with IL-13Rα to form a type II receptor complex upon IL-4 and/or IL-13 binding. This complex predominantly activates the STAT6 (Signal Transducer and Activator of Transcription 6) signaling pathway, leading to the expression of genes involved in the allergic response [2]. As IL4-Rα is shared for both IL4 and IL13-mediated signaling, inhibition of IL4Rα can block both signals simultaneously and is considered to be promising strategy to treat the diseases induced by type II inflammation [3,4]. In practice, the monoclonal antibody dupilumab targets the IL-4Rα subunit and has been highly effective treatment for type 2 inflammatory allergic diseases [5,6]. However, these monoclonal antibodies have a large molecular weight which can lead to low penetration into local tissues. In addition, although the IgG4 antibody backbone of dupilumab does not have the ability to cause complement-dependent target cell death, it still can induce unwanted target cell death by immune cells because it retains the ability to bind to antibody receptors on immune cells, albeit at a much lower level than IgG1 [7]. Based on the reported clinical manifestations, systemic administration of dupilumab typically results in an increase in serum eosinophil counts. Because hypereosinophilia can lead to potentially irreversible, life-threatening organ damage, serum eosinophils should be checked regularly [8,9]. In addition, serum sickle cell-like syndrome (SSLR), skin rash, and injection site reactions (ISR) have been reported as adverse events associated with dupilumab injection therapy, although they are less frequent in practice [[10], [11], [12], [13]]. Above all, administering regular injections to patients is not only physically inconvenient but also psychologically disturbing. Therefore, investigating the development of novel biological therapeutics with improved efficacy, fewer side effects, and no treatment discomfort is essential.

Nanobodies, derived from camelids, are the smallest naturally occurring antigen-binding fragments. They retain antigen-binding potential with a single variable domain of the heavy chain (VHH, ∼15 kDa) [14] are considered to be the 'third generation' wave of antibody [15,16]. The unique structure, consisting of four frameworks and three complement determining regions (CDRs), with four hydrophilic amino acid residues and a disulfide bond between CDR1 and CDR3, confers high solubility and stability at extreme pH or temperatures [17]. Due to their favorable properties, nanobodies hold promise for improved drug stability, delivery, and even oral or nasal administration [17,18]. Nanobodies are currently being actively developed by commercial companies and have many applications in biosensing, affinity capture, protein crystallization, imaging, and diagnostics [[19], [20], [21]]. In 2019, caplacizumab [22] was approved by the FDA for treatment thrombocytopenic purpura (aTTP) and is currently being used in clinic. In 2023, a TNFα triple nanobody was approved in Japan for treatment of arthritic conditions [23]. In addition, a very large number of tumor-targeted nanobodies or nanobody-drug conjugates are currently undergoing clinical studies for their potential to treat various solid tumors based on their high permeability [24].

The delivery of therapeutic macromolecules across biological barriers to targets without injection is highly dependent on the presence of junctional proteins between cells and the biochemical properties of the macromolecules, such as molecular weight and charge [[25], [26], [27]]. In patients with chronic rhinosinusitis with nasal polyps (CRSwNP), the expression of these tight junction proteins is significantly reduced, resulting in increased epithelial permeability [28]. Meanwhile, the rate of antibody uptake from the blood into the tissue interstitial fluid is slow and the rate of antibody clearance from the tissue is fast, resulting in antibody concentrations in the tissue being much lower than those in plasma [29]. The BC_50_ (Biodistribution coefficients 50) value of proteins for most tissues based on plasma pharmacokinetics is ∼35 kDa, giving nanobodies (∼15 kDa) or nanobody dimers (∼30 kDa) an advantage in tissue penetration over monoclonal antibodies [29,30]. Nanobodies are small protein conjugates with a highly stable structure that can pass through nasal epithelial cells, which become more permeable during inflammation, making them well suited as a platform for non-invasive therapeutic biologics with nasal access.

In the development of therapeutic biologics, proper binding to the target is the most important requirement to ensure high efficacy without side effects. In modulating this target binding, structural modelling and docking studies can make a significant contribution to the rapidly developing field of synthetic biology. These techniques can allow to predict the binding sites of a biological molecule to its target protein. Furthermore, docking studies can suggest specific modifications, such as mutations in the amino acid sequence, that can increase the binding affinity and potentially improve the therapeutic efficacy of the biologic drug. As computational methods continue to advance, accurately prediction protein structure and their interactions has become increasingly feasible, with AlphaFold2 demonstrating "experimental-level accuracy" in predicting protein structures [31,32]. In addition, improved and accessible computational approaches to predict protein-protein interactions offer highly complementary strategies for optimizing the binding affinity of biotherapeutics to their targets. Considering the labor savings, affordability, and efficiency that computational approaches bring, it is clear that the measurement of protein interaction binding affinities using continuous computer programs is an area that should be explored in various ways.

In this study, we identified a novel nanobody, H5, that targets IL-4Rα through a synthetic nanobody library with CDR diversity created via trimer oligo randomization, without animal sacrifice. Using *In-silico* methods for affinity maturation and dimerization, we enhanced H5's binding affinity to IL-4Rα by 50-fold. In human nasal epithelial cells, H5 controlled inflammation more effectively than dupilumab in the presence of IL-4 and IL-13. H5 penetrated HNEC layers, unlike dupilumab, and showed effectiveness in different cell types. These results suggest H5 and its dimer effectively inhibit inflammatory signaling in HNECs and hold promise for treating localized allergic diseases like rhinosinusitis.

## 2 Result

### 2.1 H5, a high-affinity nanobody targeting IL4Rα, discovered by synthetic library screening, effectively blocks IL-4/IL-13 signaling via apical-side treatment

**Fig. 1:**
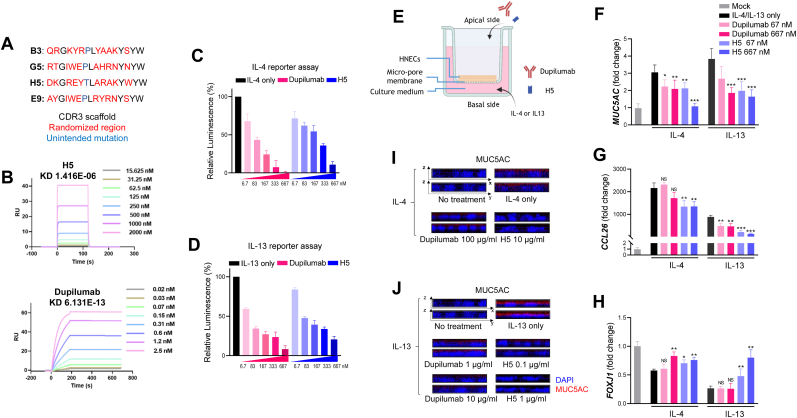
Blocking IL-4/IL-13 signaling with H5, an IL-4Rα nanobody discovered by synthetic nanobody library screening (A) Amino acid sequences of nanobody's CDR3 resulted from the screening against the IL-4Rα. Randomized region is indicated as red and unintended mutation derived during the screening processes is indicated as blue. (B) Sensograms created by Biacore T200 for binding kinetics analysis of H5 compare to dupilumab. Biotin-labeled IL-4Rα was immobilized on the sensor chip (BR100531), and 8 different doses (15.625 nM, 31.25 nM, 62.5 nM, 125 nM, 250 nM, 500 nM, 1000 nM, and 2000 nM for H5 and 0.02 nM, 0.03 nM, 0.07 nM, 0.15 nM, 0.31 nM, 0.6 nM, 1.2 nM, and 2.5 nM for dupilumab) were perfused for calculating the binding kinetics. (C–D) Dose-dependent IL-4 (upper, C)/IL-13 (lower, D) signal blocking abilities of dupilumab and H5 are measured by luciferase assay. (E) Schematic representation of the experiment for evaluating IL-4 receptor blocking function of the nanobody and dupilumab in transwell-cultured human nasal epithelial cells (HNEC). Nanobody and dupilumab were directly treated on the apical-air side of HNEC within 100 μl of media, which slightly covered the entire apical cell layer. IL-4 and IL-13 were treated in the culture media on the basal side at a concentration of 10 ng/ml each. (F–H) mRNA expression of *MUC5AC* (F), *CCL26* (G), and *FOXJ1* (H) with administration of dupilumab or H5 in IL-4 and IL-13 treated HNEC were measured by quantitative PCR. Result was summarized by the 2^ΔΔCT^ method with using GAPDH as a control for normalization. (I–J) Expressions of MUC5AC protein along the vertical layer of the HNEC under IL-4 (I) and IL-13 (J) supplemented conditions observed by confocal microscopy with z-stack setting. All data were plotted as mean ± SD, and statistical significance was assessed using Student's unpaired *t*-test: *p < 0.05, **p < 0.01, ***p < 0.001.

To screen IL4-Rα targeting nanobodies, the synthetic libraries were constructed by randomizing CDR3 with estimated diversity of 1 × 10^12^ [21]. To screen from a highly diverse pool of nanobodies, we used ribosomal display as a starting method, followed by sequential phage library screening based on two interfaces. Finally, ELISA screening using proteinaceous nanobodies identified nanobody H5, which targets the extracellular domain of human IL-4Rα (Supplementary Fig. 1). The diversity of the nanobody library pool was quantitatively assessed at each screening stage via Q-PCR. In the ELISA screening step, clones with high binding to IL-4Rα at least 1.7-fold over the negative control target, maltose-binding protein (MBP) were selected. Four different clones were identified, with H5 emerging as the final candidate due to its superior molecular binding to IL-4Rα and cytokine signaling blockade capabilities, evaluated through ELISA and reporter cell assays (Fig. 1A). Surface plasmon resonance (SPR) analysis showed that H5 has a KD of 1.42 μM, significantly lower than dupilumab's KD of 613 fM (Fig. 1B). Despite the difference in binding strength, H5 effectively inhibited STAT6-dependent luciferase expression in a dose-dependent manner, comparable to dupilumab (Fig. 1C−D), suggesting H5's similar capability to block IL-4/IL-13 dual signaling. Single-cell RNA sequencing data has shown that human nasal epithelial cells (HNECs) differentiated under liquid-liquid interface conditions closely mimic the nasal epithelium environment, reflecting mass transfer to the nasal epithelium [24]. To assess the effectiveness of H5 and dupilumab in inhibiting nasal epithelial inflammation when delivered non-invasive treatment, H5 and dupilumab were administered to the air side of HNECs (Fig. 1E). The degree of IL-4Rα receptor blockade was assessed by changing the expression of three inflammatory marker genes, *MUC5AC*, *CCL26*, and *FOXJ1*, induced by 10 μg/ml of IL-4/13 in the basolateral culture of HNECs (Fig. 1F–H). *MUC5AC* and *CCL26* are genes upregulated by IL-4 [33]and IL-13 [34] in human airway cells, while *FOXJ1* is downregulated by IL-4 [35] and IL-13 [36]. qPCR analysis showed that H5 contributed to restoring *MUC5AC* and *CCL2*6 mRNA expression to normal levels in IL-4 treated HNECs, with statistically higher restoration ability compared to dupilumab (Fig. 1F and G). For *FOXJ1* in IL-4 and IL-13 treated HNECs, gene level recovery by dupilumab treatment was observed only at a high dose, whereas recovery was observed at both doses with H5 treatment (Fig. 1H). Confocal microscopy z-stack images showed that H5 administration to the apical side of HNECs was more effective in reducing *MUC5AC* expression compared to dupilumab treatment (Fig. 1I and J).

### 2.2 Boosting H5 binding strength via *in-silico* affinity maturation and dimerization

**Fig. 2:**
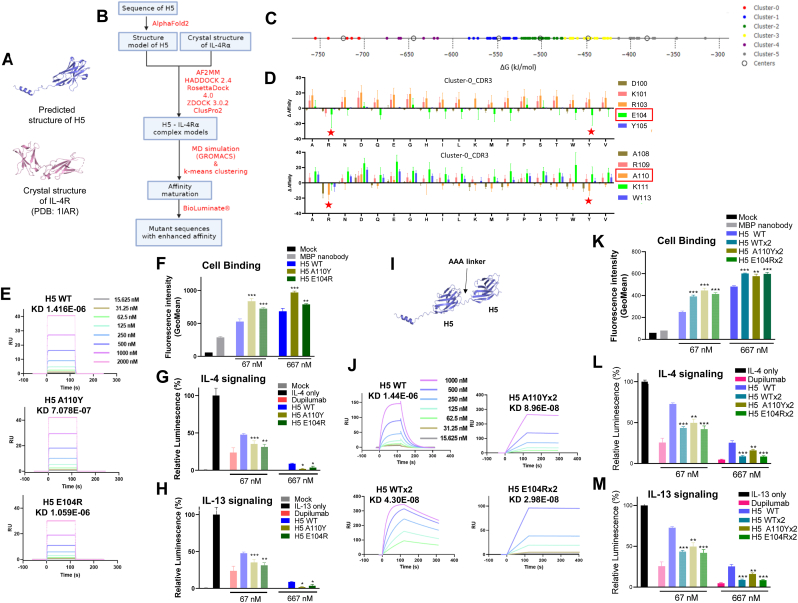
Enhancing H5 affinity through *in-silico* affinity maturation and dimerization (A) Predicted structure of nanobody H5 and crystal structure of IL-4R (PDB: 1IAR). (B) Schematic diagram of nanobody affinity maturation process. Explanations of each step are in black, and the programs used are in red. (C) All complex models are clustered into six different groups, sorted by the ΔG values derived from MD simulation. (D) Result of the residue scanning in CDR3 region of H5 by applying Schrödinger BioLuminate®. Amino acid substitutions expected to enhance binding affinity are marked with red stars. (E) Sensograms for SPR analysis of the nanobody mutants with immobilized IL-4Rα showing their binding kinetics. (F) Binding affinities of H5 and its mutant variants assessed by flow cytometry with statistical comparisons of their cell binding affinities. (G, H) Both of IL-4 (G) and IL-13 (H) signal blocking abilities of H5 and its mutant variants measured by luciferase assay with two different doses, and their receptor blocking abilities are statistically compared. (I). Representative structure of bivalent form of H5 (H5 × 2) predicted with AlphaFold2. (J) Sensograms for SPR analysis of the bivalent nanobody variants with immobilized IL-4Rα showing their binding kinetics. The process was done by iMSPR-ProX. (K) Binding affinities of H5 and its bivalent variants to IL-4Rα-expressing HEK cell assessed by flow cytometry with statistical comparisons of their cell binding affinities. (L, M) Both of IL-4 (L) and IL-13 (M) signal blocking abilities of H5 and its bivalent variants measured by luciferase assay with two different doses, and their receptor blocking abilities are statistically compared. All data were plotted as mean ± SD, and statistical significance was assessed using one-way ANOVA followed by Tukey's post hoc test: *p < 0.05, **p < 0.01, ***p < 0.001.

High binding is essential for specific effects without side effects, and most successful FDA-approved biologics have high binding [37]. For strong binding of H5, we started with the docking of the putative H5 structure predicted by AlphaFold2 with the crystallographic structure of IL-4Rα (PDB:1IAR (Supplementary Fig. 2A) shown in Fig. 2A, followed by the *in-silico* affinity maturation process (Fig. 2B). The docked decoy complex ensemble was modeled using five docking methods: AlphaFold2 multimer, Haddock 2.4, RosettaDock 4.0, ZDOCK 3.0.2, and ClusterPro 2. Given the lack of consensus on the optimal docking method, we utilized multiple methods to broaden the conformational search space [29]. We used GROMACS to reassess the decoy quality based on the binding free energy estimated through the MM-PBSA method. Among the 95 decoys evaluated, the top six candidates with the highest binding free energies were selected for mutagenesis (Supplementary Fig. 2B). These candidates were identified as the best cluster by k-means clustering (Fig. 2C). A saturation approach was used for mutagenesis, considering all 20 natural amino acids at positions relevant to the CDR3 region in H5 using BioLuminate®. Amino acid substitutions predicted to significantly increase binding affinity were highlighted with red stars (Fig. 2D). The mutated H5 increased the number of hydrogen bonds when interacting with IL-4Rα (Supplementary Fig. 2D). Amino acid substitutions in H5^A110Y^ and H5^E104R^ enhanced the binding affinity of H5 in the ELISA assay (Supplementary Fig. 3A). SPR analysis showed that H5 mutants, H5^A110Y^ and H5^E104R^, had enhanced binding affinity for IL-4Rα (708 nM and 1.03 μM, respectively) compared to H5^WT^ (1.42 μM) (Fig. 2E–Supplementary Table 1). The mutants also showed enhanced affinity for IL-4Rα-overexpressing cells (Fig. 2F) and improved blockade of IL-4 (Fig. 2G) and IL-13 (Fig. 2H) signaling at 30 μg/ml of cytokine treatment.

Divalent nanobodies have higher binding affinity for their targets [38,39]. We combined H5 nanobodies with a tri-alanine linker to create dimeric structures, generating bivalent nanobody H5 (H5 × 2) with affinity-enhancing mutations. As suggested by the similarity in conformation between the crystal structure of IL4-IL4R complex (Supplementary Fig. 2A) and the predicted complex model of IL4R-H5, competitive binding experiments via BLI binning assay showed that H5 and IL4 compete for binding to IL4R (Supplementary Fig. 2B). All divalent nanobodies showed significantly enhanced binding in the ELISA assay (Supplementary Fig. 3B), and SPR results explained the enhanced binding after dimerization. All bivalent nanobodies H5^WT^ × 2, H5^A110Y^ × 2, and H5^E104R^ × 2 showed enhanced binding affinity for IL-4Rα (43.0 nM, 89.6 nM, and 29.8 nM, respectively) compared to H5 monomer (1.42 μM) (Fig. 2I and J, Supplementary Table 1). The bivalent nanobodies also showed enhanced binding affinity to IL-4Rα overexpressing stable cell lines compared to H5 (Fig. 2K) and improved ability to block IL-4 (Fig. 2L) and IL-13 (Fig. 2M) signaling at 30 μg/ml cytokine treatment.

### 2.3 Nanobody size and cellular penetration

**Fig. 3:**
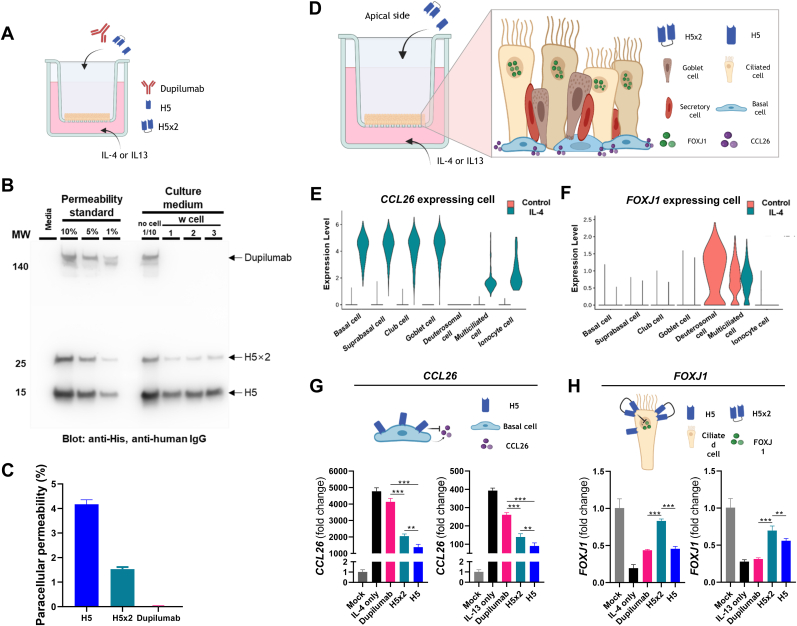
Nanobody penetration and its effects on cells at varying depths (A) Schematic representation for experiment evaluating paracellular permeability of H5, H5 × 2 and dupilumab. The molecules were administered on the apical-air side of HNECs with concentration of 100 μg/ml each, and IL-4/IL-13 were treated in the culture media on the basal side with concentration of 10 ng/ml each. (B) Western blot of the culture medium containing cell-permeated monovalent H5, bivalent H5, and dupilumab, indicating the paracellular permeability of the molecules. (C) Paracellular permeabilities (basal side permeable amount/total apical treated amount*100 (%)) of monovalent H5, bivalent H5, and dupilumab were calculated by each band intensity. (D) Schematic representation of experiment for evaluating receptor blocking function of the monovalent and bivalent nanobodies as well as indicating the cell composition of the HNEC. Both nanobodies were directly treated on the apical side of HNEC, and 10 ng/ml of IL-4/IL-13 were treated in culture medium on the basal side. (E, F) Violin plots displaying the expression levels of *CCL26* (E) and *FOXJ1* (F) genes in distinct subsets of HNEC cells under normal and IL-4 treated conditions, as determined through single-cell sequencing analysis. (G, H) Expression of *CCL26* (G) and *FOXJ1* (H) with administration of the nanobody variants in IL-4 (G) and IL-13 (H) supplemented condition in HNEC detected by quantitative PCR.

To demonstrate the cell membrane permeability of H5 and H5 × 2, HNECs cultured at the air-liquid interface were treated with 100 μg/ml of H5, H5 × 2, and dupilumab on the air side and incubated for 72 h (Fig. 3A). The basolateral culture medias were analyzed by western blotting to identify permeable molecules (Fig. 3B). The band intensity comparison indicated cell membrane permeability (Fig. 3C). Dupilumab showed no permeability, while H5 and H5 × 2 showed permeability of about 4 % and 1 %, respectively.

To determine whether monovalent or bivalent nanobody inhibits IL-4 or IL-13 signaling depending on cellular location, we observed the effect on two inflammatory markers expressed in different cells. We treated the apical side of HNECs with 333 nM of the nanobody and compared it to the basal side of HNECs treated with 20 μg/ml of IL-4/IL-13 (Fig. 3D). The expression of *CCL26*, a gene expressed in basal cells [31] (Fig. 3E, Supplementary Fig. 5A), was more downregulated in monovalent nanobody-treated samples in IL-4 and IL-13-treated HNECs (Fig. 3G). In contrast, *FOXJ1*, a gene expressed in ciliated cells [32] (Fig. 3F, Supplementary Fig. 5B), was more upregulated in bivalent nanobody-treated samples in IL-4 and IL-13-treated HNECs (Fig. 3H). These results suggest differences in binding strength and permeability between monovalent and divalent nanobodies, with divalent nanobodies affecting easily accessible cells and monovalent nanobodies impacting less accessible cells near the base.

## 3 Discussion

The key mechanism by which dupilumab inhibits IL-4 or IL-13 signaling is by binding to the target protein IL-4Rα and interfering with the binding of IL-4 or IL-13. Antibody structure is not necessary for the ability of dupilumab to inhibit IL-4/IL-13 signaling, as it is composed of the least immunogenic Fc of IgG4. Considering that protein size plays the most important role in penetrating nasal epithelial cells, smaller target-binding modules would be more effective in developing non-invasive therapeutic pathways [25,40]. A form of medicine that efficiently penetrates between cells to bind to key therapeutic targets could pave the way for new non-invasive treatments that do not require injections. Since most allergic diseases typically manifest as localized symptoms, the development of topical route of administration treatments for allergic diseases could circumvent the systemic effects and side effects of injectable drugs [13]. In this context, nanobodies offer a promising non-invasive treatment for rhinosinusitis, an inflammatory disorder in the nasal cavity. These small protein-based drugs can be delivered directly to the nose, potentially increasing treatment efficacy and favorability while avoiding systemic side effects commonly associated with traditional treatments.

The expression of tight junction proteins in nasal epithelial cells is significantly reduced in patients with rhinitis and nasal polyps [4,25,41]. Treatment of HNECs with IL-4 and IL-13, which are highly concentrated in the nasal epithelium of rhinitis patients, decreases the expression of *ZO-1* and *Occludin*, components of tight junctions (Supplementary Figs. 4A and B) [28]. These conditions suggest that small-sized proteins can be easily transported in nasal epithelial cells of rhinitis patients, facilitating non-invasive therapeutics. It is noteworthy that nanobody H5, applied to the apical air side of HNECs of rhinitis patients, shows statistically superior performance in blocking the dual signaling of IL-4/IL-13 compared to dupilumab. H5 significantly restores the expression of *MUC5AC*, *CCL26*, and *FOXJ1* genes to normal levels (Fig. 1F–H, Fig. 3g-h). Most importantly, H5 (KD 1.42 μM) showed higher potency compared to dupilumab (KD 613 fM), emphasizing the importance of small size in non-invasive therapy. The difference in affected cells depending on the monomeric or dimer is also significant. The bivalent variant H5 × 2, with high affinity for IL-4Rα, effectively inhibited IL-4/IL-13 signaling in cells in the upper nasal epithelial zone, while the monovalent form H5 showed significant efficacy in deeper nasal epithelial layers, despite a relatively low binding affinity compared to H5 × 2.

Despite the significant increase in binding affinity when transformed into the divalent form, monomeric nanobodies with very high binding affinity may be more effective for ensuring therapeutic effects reach all cells in different anatomical locations. The percentage of basal cells in patients with nasal polyps is significantly higher than in healthy individuals [42], emphasizing the need for monomeric nanobodies with very high binding affinity.

In this study, *in-silico* methods identified mutations that enhance the binding capacity of nanobodies. Given that the time required for simulation of protein structure prediction is proportional to the size of the protein, small, single-chain nanobodies have a significant time-saving advantage over full-length antibodies [30]. Structural analysis of the mutant-IL4Rα complex model revealed that the number of hydrogen bonds between the nanobody and IL-4Rα increased from 7 for H5^WT^ to 10 for H5^A110Y^ and 11 for H5^E104R^ (Supplementary Fig. 2D, red dotted line). The increased binding affinity to the target molecule was presumably due to the increased number of hydrogen bonds. Although the divalent formation of the nanobody contributed more to the binding affinity compared to the *in-silico* affinity maturation process, the synergistic effect of the two strategies resulted in the nanobody with the highest molecular binding affinity (H5^E104R^ × 2 Supplementary Table 1). Since the binding enhancement in this study was specific to CDR3, applying it to CDR1 and CDR2 could induce even higher binding enhancement.

Many allergic inflammatory associated with type 2 immune responses primarily manifest at localized sites; therefore, non-invasive treatments such as nasal sprays, topical applications, or skin applications offer significant advantages in reducing systemic side effects. The nanobody-based approach we propose effectively inhibits type 2 inflammation, making it a promising platform for treating various localized autoimmune diseases. Considering advancements in non-invasive drug delivery systems, developing such systems tailored for nanobodies could significantly enhance therapeutic efficacy and precision. Future studies should focus on optimizing these delivery mechanisms to maximize the penetration and retention of nanobodies in targeted tissues, leading to highly effective, patient-friendly treatments for allergic rhinitis and other localized allergic conditions.

In conclusion, the IL-4Rα-targeting nanobody H5 presents a promising non-invasive treatment strategy for allergic rhinitis. Our findings highlight the potential of combining nanobodies with advanced delivery systems to enhance therapeutic outcomes. Continued research in this area will be crucial for translating these findings into clinical applications, offering new hope for patients with localized allergic diseases.

## 4 Materials and methods

### 4.1 Cells and materials

HEK293T and HEK293 cell line were purchased from the Korea Cell Line Bank. Those cells were cultured in DMEM high glucose media (Gibco, 11995-065) supplemented with 10 % FBS (Gibco, 26140-079) and penicillin/streptomycin (Gibco, 15140-122) at 37 °C, 5 % CO_2_. The used materials are listed in supplementary materials with detailed information.

### 4.2 Construction of randomized convex scaffold DNA library

pRDV containing Sb-convex (Addgene, 132696) was chosen as a template for the PCR. FW_c_for and Link2_c_rev primers were used for amplifying the former region of the convex scaffold which contains CDR1 and CDR2 regions. Link2_c_for, FW_c_rev, and CDR3_c primers were used for amplifying the latter region of the convex scaffold with randomization of the CDR3 region. The randomized primer CDR3_c includes 10 different trinucleotide residues, nine of them (111) are enriched with A, S, T, N, Y (10.6 % each), contains D, E, Q, R, K, H, W (5 % each), and harbors few apolar amino acids F, M, V, I, L, G (2 % each)^31^. One another trinucleotide residue (222) lacks amino acid D and A since those two are underrepresented at the end of β-sheets^32^. BbsI restriction site exists at the both ends of the former (scaffold) and latter (randomized) convex fragments, they were restricted and were subsequently ligated to be formed convex library which has a diversity of $3.17\times 10^{12}$.

### 4.3 Screening of nanobody H5 using synthetic library

In the first screening process ribosome display, RNA of the synthetic nanobody library pool was translated *in-vitro*, and molecules bound to the immobilized IL-4Rα were collected. The RNA elute was synthesized to cDNA and successively amplified to be cloned into the phagemid vector. Phage containing library pool was produced to be used for 2 rounds of phage display, and the phagemids containing nanobody gene which is able to bind to the IL-4Rα were cloned into expression vector. Single colonies of BL21 *E. coli* containing each expression plasmid were evaluated by ELISA to choose top hits that have specific binding affinity against the IL-4Rα.

### 4.4 Production and purification of the nanobodies

Nanobody clone in the expression vector (pSBinit) was transformed into the *E. coli* BL21, and cultured in 50 ml of TB medium at 22 °C, 160 rpm for overnight with 25 μg/ml of chloramphenicol and 0.02 % of arabinose supplement. The overnight culture was centrifuged and the pallet was resuspended with 4 ml of the periplasmic extraction buffer (20 % sucrose, 50 mM Tris pH 8.0, 0.05 mM EDTA, 0.5 μg/ml lysozyme) in the ice for 30 min. The resuspension was centrifuged and successively its supernatant was diluted in 36 ml of TBS pH 8.0. 1 ml of TALON®Superflow™ (Cytiva, 28957502) slurry was pre-equilibrated with TBS pH 8.0. The periplasmic supernatant was incubated with 1 ml of slurry in 4 °C for 1 h with gentle rotation. After the binding, the beads were washed with the washing buffer (50 mM Tris pH 8.0, 300 mM NaCl, and 5 mM imidazole) for 3 times, and then the nanobody was eluted with the elution buffer (50 mM Tris pH 8.0, 300 mM NaCl, and 200 mM imidazole). The eluted nanobody was undergone to the buffer change by using Slide-A-Lyzer® Dialysis Cassettte (Thermo, 66330) for overnight.

### 4.5 STAT6-luciferase reporter system for measuring IL-4R inhibition

The HEK293 cell line was developed by stable expression of human STAT6 (Addgene, 81950) by puromycin-resistant lentivirus system, and pSTAT6-induced luciferase (Addgene, 35554) by blasticidin-resistant system, so that luciferases can be stably expressed under control of STAT6 response from the IL-4 signaling [43]. ${5\times 10}^{4}$ of reporter cells were seeded into each well of 96-well cell culture plate and cultured with IL-4 (Enzynomics, C008)/IL-13 (Enzynomics, C009) and one of the dupilumab or nanobody, with DMEM, at 37 °C and 5 % CO_2_ for 24 h. After removing the medium, the cells were resuspended and washed with PBS, subsequently lysed under 20 μl of lysis buffer (25 mM Tris pH 8.0, 4 mM EGTA, 10 % glycerol, and 10 μl of Triton X-100 in 1 ml of DW). The cell lysis was transferred to the white 96-well plate, 50 μl of luciferase substrate (Promega, E1501) was added. Random luminescence unit was measured by SpectraMax®M5 luminometer (Molecular Devices).

### 4.6 Surface plasmon resonance (SPR)

IL-4Rα was immobilized on the sensor chip (BR100531 for Biacore T200 and HC1000 M for iMSPR-ProX) with 200 nM of running buffer (PBS, pH 7.4). 8 different concentrations (15.625 nM, 31.25 nM, 62.5 nM, 125 nM, 250 nM, 500 nM, 1000 nM, 2000 nM) nanobodies were undergone to the binding analysis for Biacore T200, 5 different concentrations (31.25 nM, 62.5 nM, 125 nM, 250 nM, 500 nM, 1000 nM) for iMSPR-ProX, with 50 μg/ml of flow rate. Regeneration of the nanobodies was done with generation buffer (10 mM glycine-HCl, pH 3.0) with 50 μg/ml of flow rate. In order to compute equilibrium dissociation rate constant (k_a_, k_d_, and k_D_), 1:1 kinetic binding model was used.

### 4.7 *In-silico* affinity maturation

A putative structure of H5 was predicted by using ColabFold v1.5.2, an AlphaFold2 server hosted on Google Colab [44]. The putative structure of the H5 was then docked to a crystallographic structure of IL-4Rα downloaded from the Protein Data Bank (PDB: 1IAR) to produce an ensemble of decoy complexes (Supplementary Figs. S2C–D). Before the docking, the structures were undergone to protein preparation to add hydrogen atoms and to exclude water molecules. The complex decoys were modeled utilizing 5 different docking methods, namely AlphaFold2 multimer [44], HADDOCK 2.4 [45], RosettaDock 4.0 [46], ZDOCK 3.0.2 [47], and ClusPro 2 [48] to maximize the conformational search space and hit rate. Among the decoy complexes, a group of candidates with the highest binding free energies were selected to undergo *in-silico* mutagenesis. Since determining if a decoy complex is near-native based on its docking score is unsolved issue, we re-evaluated the quality of decoys based on theirs binding free energies estimated via Molecular Mechanics-Poisson Boltzman Surface Are (MM-PBSA) [49] method using GROMACS molecular dynamics simulation package. To account for estimation errors in various steps, we opted to select a group of decoys instead of just the best one for the subsequent mutagenesis. A group of candidates with the lowest binding free energies were identified using the k-means clustering (Supplementary Fig. S2A). A saturation approach, where all 20 natural amino acids were considered in positions involved in CDR3 region, was employed in the mutagenesis using the residue scanning functionality of BioLuminate®.

### 4.8 Culture of primary human nasal epithelial cells (HNEC)

This study was approved by the Institutional Review Board of Yonsei University College of Medicine (4–2016-1153, 4-2021-0573). The HNEC cells were extracted from patients with chronic rhinosinusitis and nasal polyps. The primary HNEC in the second passage were placed in a culture medium that consisted of a 1:1 blend of basal epithelial growth medium. The HNEC was maintained in the Transwell®-clear (Costar Co.). The cells were cultured for three days while submerged. Afterwards, the apical culture medium was removed to create an air-liquid interface (ALI). For each experiment, the fully differentiated HNEC was applied two weeks after the establishment of the ALI.

### 4.9 Immunofluorescence staining and image analysis

HNE cells from transwell were washed with PBS and fixed with 500 μl of 4 % paraformaldehyde (Biosesang, P2031) for 30 min at room temperature. After 3 times of washing with PBS, the fixed cells were permeabilized with 0.5 % Triton X-100, followed by blocking with 50 μl of 1 % BSA for 30 min. The HNE cells were then incubated with 100 μl (1:200) of FOXJ1 primary antibody (GeneTex, GTX114408) or MUC5AC primary antibody (Santacruz biotechnology, sc-21701) for 1 h and subsequently incubated with 100 μl of (1:1000) mouse anti-rabbit FITC (Jackson Immunoresearch, 211-095-109) and goat anti-mouse 647 (Jackson Immunoresearch, 115-605-003) for 30 min and subsequently washed with PBS for three times. The cells were followed by photographing under the Zeiss LSM 700 using the parameter setting of 405 nm and 488 nm lasers and Z-stack multidimensional acquisition function was applied. The images of Z-stack slices were obtained by setting up the interval at 8 μm.

### 4.10 qPCR analysis of inflammatory markers and tight junction genes in HNEC

mRNA in the HNEC was extracted by Trizol (Favorgen, FATRR001), then phase separation of the extract was done by Chloroform (Sigma, C2432). The upper aqueous phase was collected and mRNA was precipitated by isopropanol. The harvested mRNA was undergone to reverse transcription by SuperiorScript III reverse transcriptase kit (Enzynomics, RT006). For the quantitative PCR, QuantStudio 3 Real-time PCR instrument (Applied Biosystems) was used with AccuPower® 2X Greenstar qPCR master mix (Bioneer, K-6251). Sequences of the used PCR primers are indicated in the supplementary materials.

### 4.11 Single cell analysis HNEC

We used a LUNA-FL™ Automated Fluorescence Cell Counter from Logos Biosystems to prepare the cells for scRNAseq. This was done in accordance with the 10× Genomics Single Cell Protocols Cell Preparation Guide and the Guidelines for Optimal Sample Preparation flowchart (Documents CG00053 and CG000126, respectively). The preparation of libraries was carried out with a Chromium Controller in accordance with the 10 × Single Cell 3’ v2, v3 protocol as directed by 10× Genomics. Cell Ranger v3.1.0 (10 × Genomics) pipeline was applied for converting raw sequencing data into the FASTQ files, and Illumina base cell files were converted into the FASTQ format using the “mkfastq” command. To analyze 3′ gene expression library data, we utilized the Cell Ranger v3.1.0 (10X Genomics) pipeline to convert the raw sequencing data into FASTQ files. Illumina base cell files were converted into the FASTQ format using the “mkfastq” command. The STAR aligner (v2.5.1b) was used to align sequencing reads to the GRCh38 genome reference. Gene expression profiling of each cell was carried out through the application of unique molecular identifiers (UMIs) and the 10 × cell barcodes. Sequencing reads were aligned to the GRCh38 genome reference using the STAR aligner (v2.5.1b), and gene expression profiling of each cell was performed by applying unique molecular identifiers (UMIs) and the 10 × cell barcodes. We imported raw count matrices from the 10x genomics pipeline into Seurat 3.1.0. For downstream analysis, we used raw counts for all genes expressed in at least three cells and all cells with at least 200 detected genes. The cells were successively clustered into each group according to their gene expression.

### 4.12 Cellular permeability analysis

Fully differentiated HNEC after the ALI establishment was applied for the experiment. The monomer H5, bivalent H5, and dupilumab were administered on the apical-air side of the HNE with concentration of 100 μg/ml in 100 μl of media, and 20 ng/ml of IL-4/IL-13 were treated in the media on the basal side. After the 72 h of incubation at 37 °C, 5 % CO_2_, culture media was undergone to the western blot analysis. Mouse anti-His tag monoclonal antibody (Invitrogen, MA1-21315) was used as a primary antibody, Goat anti-mouse IgG-HRP (Jackson Immunoresearch, 115-034-003) and goat anti-human IgG-HRP (Jackson Immunoresearch, 109-035-003) were used as secondary antibody for nanobodies and dupilumab respectively.

### 4.13 Statistical analysis

For all experiments, data are presented as mean ± standard deviation (SD). Statistical significance was assessed using Student's unpaired *t*-test, unless otherwise specified. For comparisons involving more than two groups, one-way ANOVA followed by Tukey's post hoc test was performed. A p-value of less than 0.05 was considered statistically significant. All analyses were conducted using GraphPad Prism software (version 8).

## CRediT authorship contribution statement

**Taeyoung Ahn:** Writing – original draft, Methodology, Investigation, Formal analysis, Data curation. **Dong Hyuk Lee:** Validation, Methodology, Investigation. **GeunAh Kim:** Formal analysis. **JiHyun Kim:** Formal analysis. **Joon-Sang Park:** Validation, Supervision, Software, Data curation. **Hyung-Ju Cho:** Resources, Investigation, Formal analysis, Data curation. **Joo Young Kim:** Writing – review & editing, Writing – original draft, Supervision, Project administration, Funding acquisition, Conceptualization.

## Declaration of competing interest

The authors declare that they have no known competing financial interests or personal relationships that could have appeared to influence the work reported in this paper.

## Data Availability

No data was used for the research described in the article.
