# Supplementary material for: Non-invasive strategy: Developing a topical IL-4Rα-specific nanobody for the treatment of allergic airway diseases
Source: Mater Today Bio. 2024 Jul 8;27:101148. doi: 10.1016/j.mtbio.2024.101148 (PMC11301380; doi:10.1016/j.mtbio.2024.101148)
Supplement: Multimedia component 1 [file mmc1.pptx]

## Slide 1
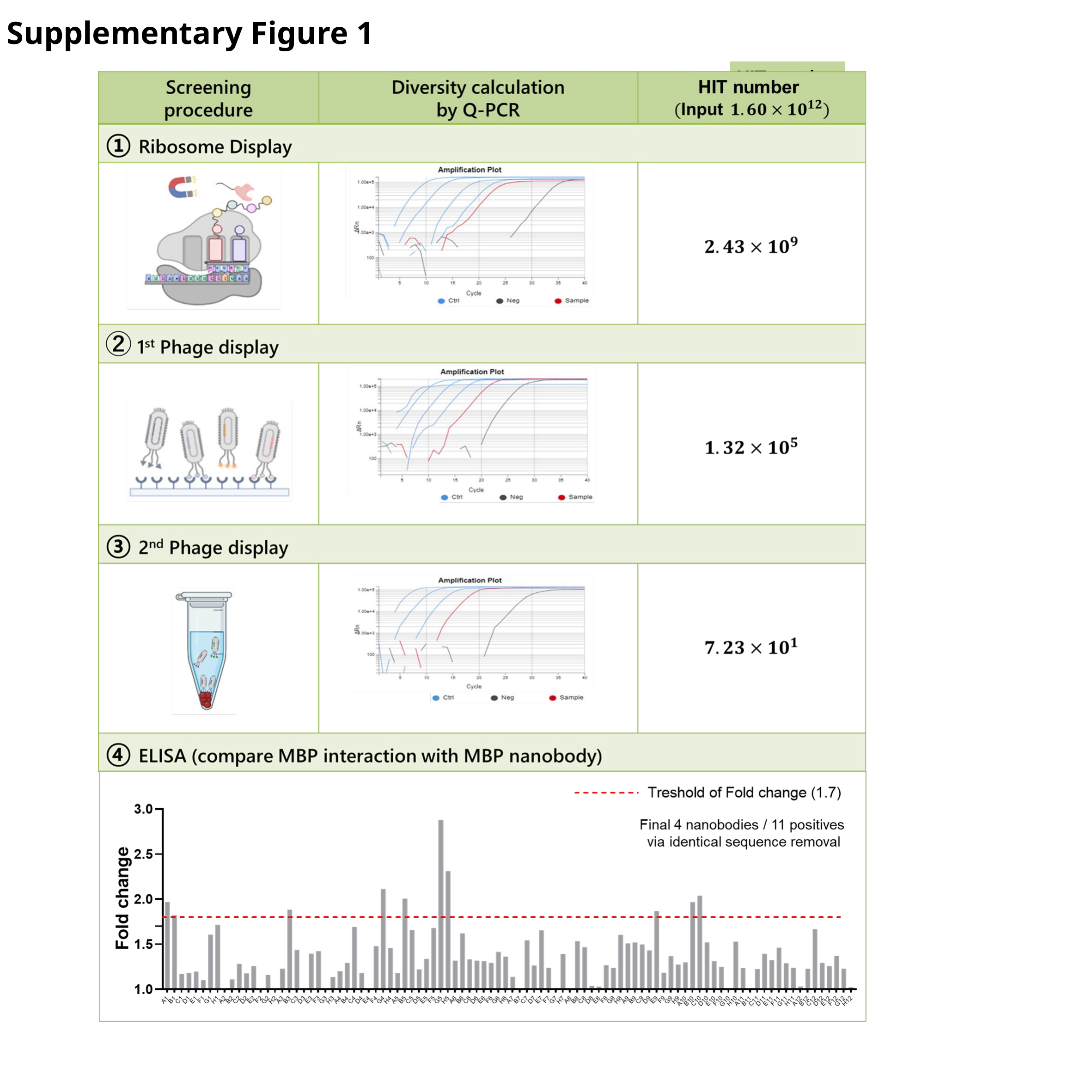

Supplementary Figure 1
HIT number

## Slide 2
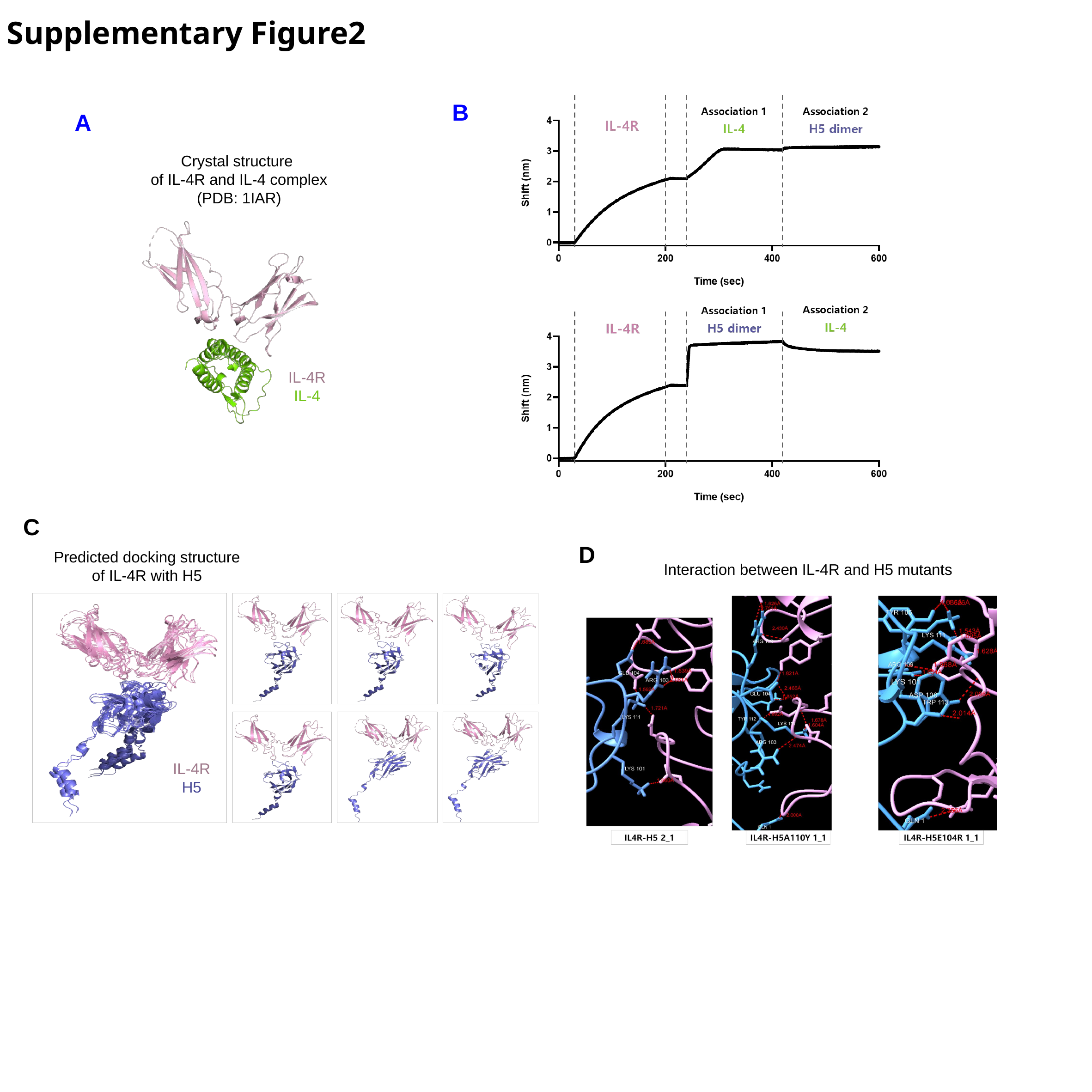

Supplementary Figure2
B
A
Crystal structure
of IL-4R and IL-4 complex
(PDB: 1IAR)
IL-4R
IL-4
C
D
Interaction between IL-4R and H5 mutants
Predicted docking structure of IL-4R with H5
IL-4R
H5

## Slide 3
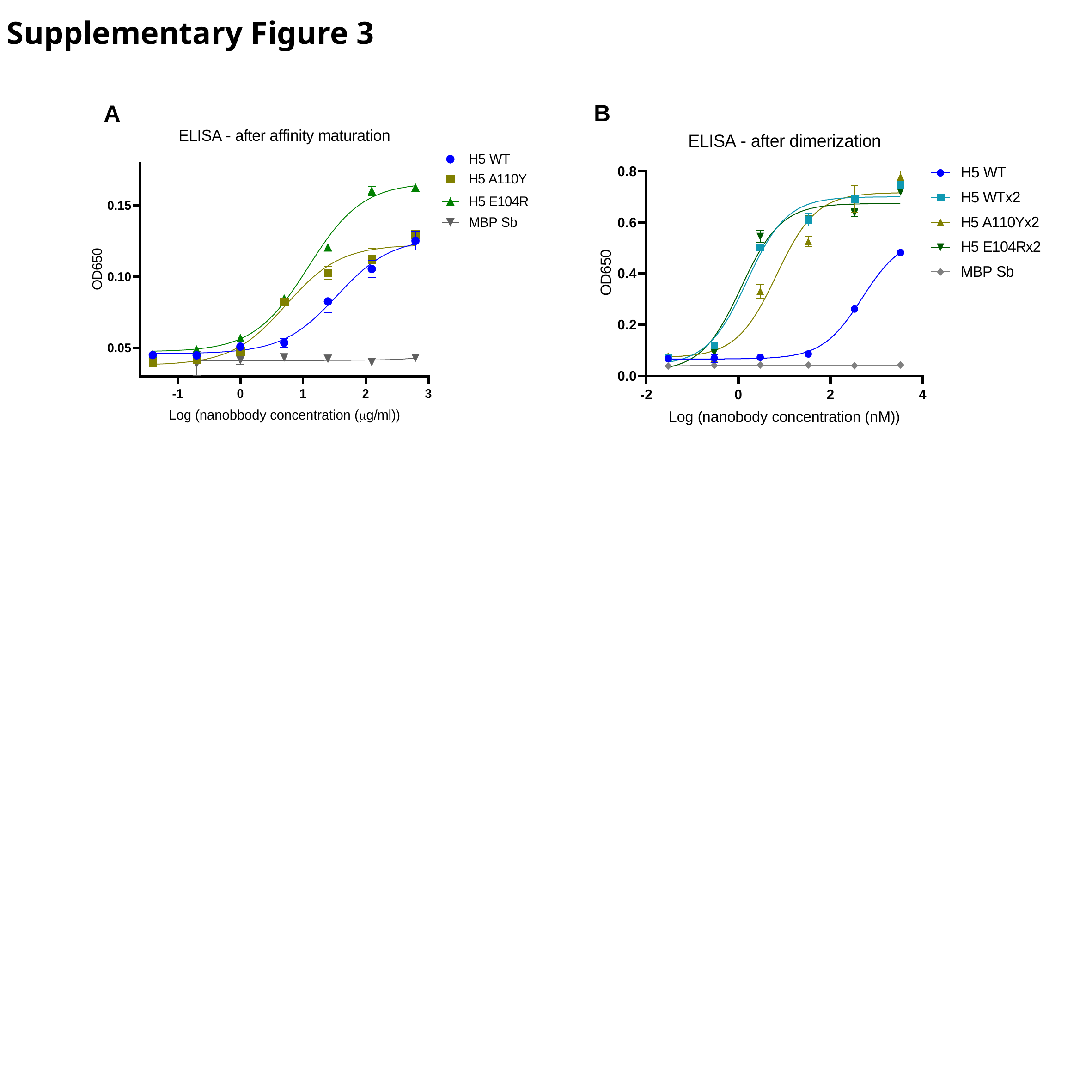

Supplementary Figure 3
B
A

## Slide 4
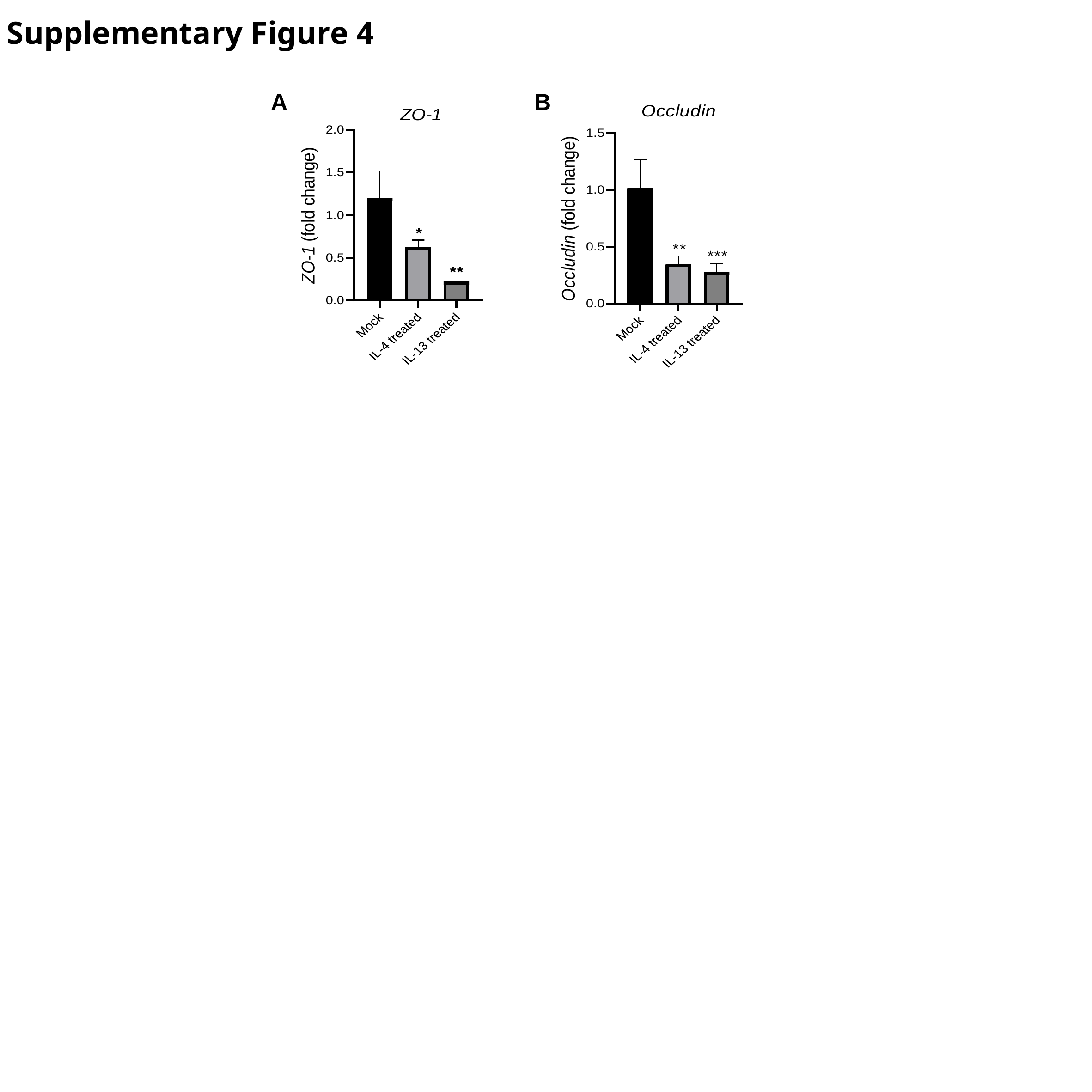

Supplementary Figure 4
A
B

## Slide 5
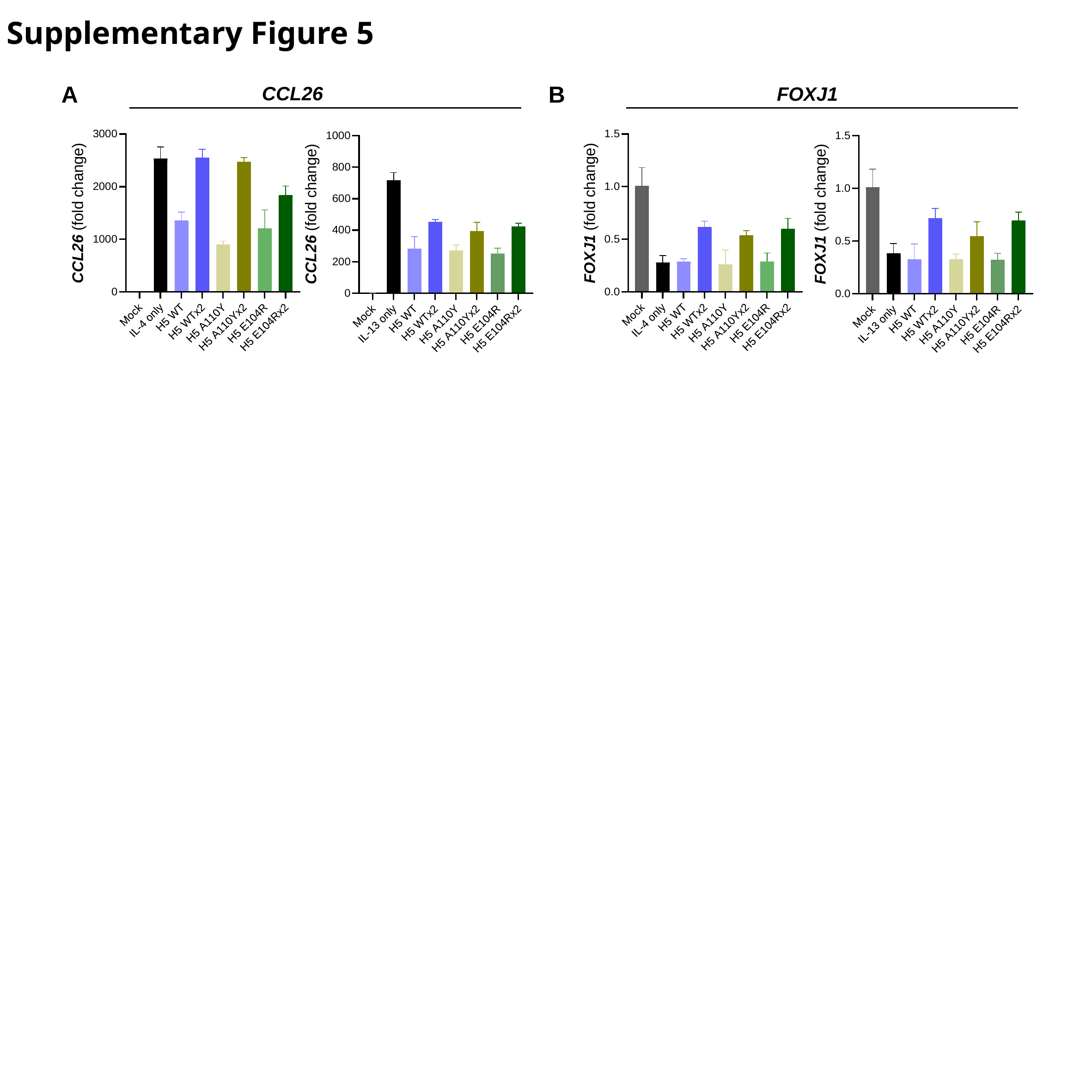

Supplementary Figure 5
A
B
CCL26
FOXJ1

## Slide 6
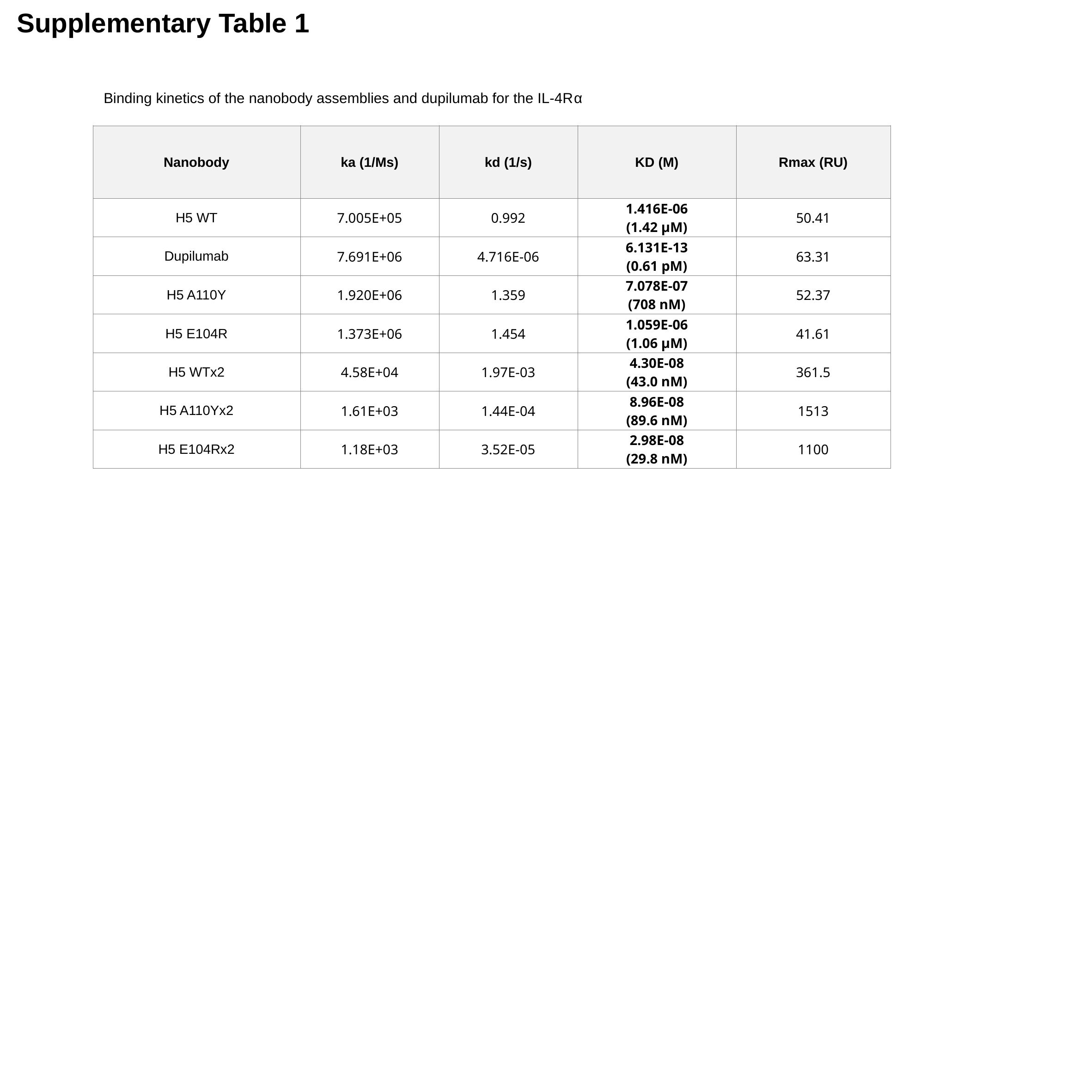

Supplementary Table 1
Binding kinetics of the nanobody assemblies and dupilumab for the IL-4Rα
| Nanobody | ka (1/Ms) | kd (1/s) | KD (M) | Rmax (RU) |
| --- | --- | --- | --- | --- |
| H5 WT | 7.005E+05 | 0.992 | 1.416E-06 (1.42 µM) | 50.41 |
| Dupilumab | 7.691E+06 | 4.716E-06 | 6.131E-13 (0.61 pM) | 63.31 |
| H5 A110Y | 1.920E+06 | 1.359 | 7.078E-07 (708 nM) | 52.37 |
| H5 E104R | 1.373E+06 | 1.454 | 1.059E-06 (1.06 µM) | 41.61 |
| H5 WTx2 | 4.58E+04 | 1.97E-03 | 4.30E-08 (43.0 nM) | 361.5 |
| H5 A110Yx2 | 1.61E+03 | 1.44E-04 | 8.96E-08 (89.6 nM) | 1513 |
| H5 E104Rx2 | 1.18E+03 | 3.52E-05 | 2.98E-08 (29.8 nM) | 1100 |

## Slide 7
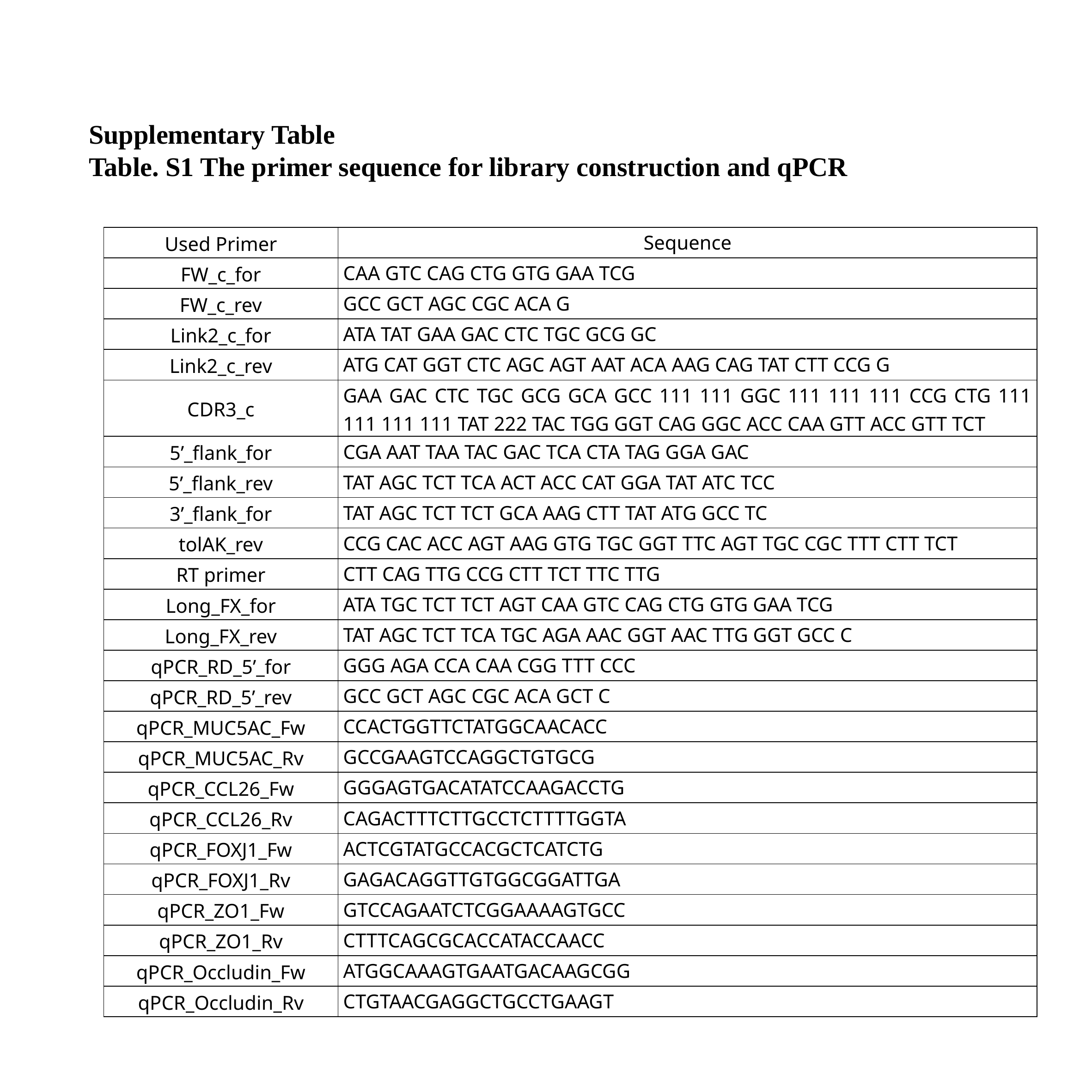

Supplementary Table
Table. S1 The primer sequence for library construction and qPCR
| Used Primer | Sequence |
| --- | --- |
| FW\_c\_for | CAA GTC CAG CTG GTG GAA TCG |
| FW\_c\_rev | GCC GCT AGC CGC ACA G |
| Link2\_c\_for | ATA TAT GAA GAC CTC TGC GCG GC |
| Link2\_c\_rev | ATG CAT GGT CTC AGC AGT AAT ACA AAG CAG TAT CTT CCG G |
| CDR3\_c | GAA GAC CTC TGC GCG GCA GCC 111 111 GGC 111 111 111 CCG CTG 111 111 111 111 TAT 222 TAC TGG GGT CAG GGC ACC CAA GTT ACC GTT TCT |
| 5’\_flank\_for | CGA AAT TAA TAC GAC TCA CTA TAG GGA GAC |
| 5’\_flank\_rev | TAT AGC TCT TCA ACT ACC CAT GGA TAT ATC TCC |
| 3’\_flank\_for | TAT AGC TCT TCT GCA AAG CTT TAT ATG GCC TC |
| tolAK\_rev | CCG CAC ACC AGT AAG GTG TGC GGT TTC AGT TGC CGC TTT CTT TCT |
| RT primer | CTT CAG TTG CCG CTT TCT TTC TTG |
| Long\_FX\_for | ATA TGC TCT TCT AGT CAA GTC CAG CTG GTG GAA TCG |
| Long\_FX\_rev | TAT AGC TCT TCA TGC AGA AAC GGT AAC TTG GGT GCC C |
| qPCR\_RD\_5’\_for | GGG AGA CCA CAA CGG TTT CCC |
| qPCR\_RD\_5’\_rev | GCC GCT AGC CGC ACA GCT C |
| qPCR\_MUC5AC\_Fw | CCACTGGTTCTATGGCAACACC |
| qPCR\_MUC5AC\_Rv | GCCGAAGTCCAGGCTGTGCG |
| qPCR\_CCL26\_Fw | GGGAGTGACATATCCAAGACCTG |
| qPCR\_CCL26\_Rv | CAGACTTTCTTGCCTCTTTTGGTA |
| qPCR\_FOXJ1\_Fw | ACTCGTATGCCACGCTCATCTG |
| qPCR\_FOXJ1\_Rv | GAGACAGGTTGTGGCGGATTGA |
| qPCR\_ZO1\_Fw | GTCCAGAATCTCGGAAAAGTGCC |
| qPCR\_ZO1\_Rv | CTTTCAGCGCACCATACCAACC |
| qPCR\_Occludin\_Fw | ATGGCAAAGTGAATGACAAGCGG |
| qPCR\_Occludin\_Rv | CTGTAACGAGGCTGCCTGAAGT |
